# Plasmonic trapping of nanoparticles by metaholograms

**DOI:** 10.1038/s41598-017-11301-1

**Published:** 2017-09-05

**Authors:** Guanghao Rui, Yanbao Ma, Bing Gu, Qiwen Zhan, Yiping Cui

**Affiliations:** 1 Advanced Photonics Center, Southeast University, Nanjing, 210096 Jiangsu China; 2 Department of Electro-Optics and Photonics, University of Dayton, 300 College Park, Dayton, OH 45469 USA

## Abstract

Manipulation of nanoparticles in solution is of great importance for a wide range of applications in biomedical, environmental, and material sciences. In this work, we present a novel plasmonic tweezers based on metahologram. We show that various kinds of nanoparticles can be stably trapped in a surface plasmon (SP) standing wave generated by the constructive interference between two coherent focusing SPs. The absence of the axial scattering force and the enhanced gradient force enable to avoid overheating effect while maintaining mechanical stability even under the resonant condition of the metallic nanoparticle. The work illustrates the potential of such plasmonic tweezers for further development in lab-on-a-chip devices.

## Introduction

In 1986, Ashkin and colleagues reported the first observation of a stable three-dimensional optical trap, or optical tweezers, created using radiation pressure from a single laser beam^1^. An optical tweezers is a scientific instrument used to apply piconewton-sized forces and make precise measurements on a scale of roughly one micron. It allows scientists to make detailed manipulations and measurements on tiny objects, and thus is an important tool in biophysics^2^. In recent years, nanometer-sized metallic particles have attracted increasing attentions owing to their special chemical and physical properties and extensive applications in various areas^3, 4^. Due to the noncontact and holding nature, optical tweezes is highly desirable to apply those exotic properties in free solution and in absence of substrate. The movement behavior of nanoparticles in an optical tweezers is determined by the competition of gradient force and scattering force, which are caused by the intensity inhomogeneity and the scattering/absorption of particles, respectively. Therefore, stable optical trap comes down to mitigating the adverse effect of scattering force to the trapping stability. To increase the trapping efficiency of metallic nanoparticle, radial polarization has been proposed to replace the conventional scalar beam as the illumination in optical tweezers^5, 6^. The advantage of using radial polarization is the absence of axial radiation force, which is due to the special focusing properties of cylindrical vector beam. As one type of scattering force, spin curl force arises from the vector nature of the light usually is negligible for optical beams with spatially homogeneous states of polarization (SOP). However, it cannot be neglected for vectorial optical field with spatially variant SOPs^7, 8^. As the trapping laser approaches the resonant wavelength of the metallic nanoparticle, the scattering force increases rapidly and the particle would be strongly pushed away from the light source. With the development of the optical engineering, generation of optical field with inhomogeneous spatial distribution in terms of phase, amplitude and polarization becomes possible, providing more degrees of freedom to tailor the optical force^9–12^. For example, it has been reported that negative scattering force pointing against the power flow can be generated with Bessel beams, which shows the potential to achieve stable manipulation of resonant metallic nanoparticle by the subtle balance between gradient force and scattering force^13–16^. Besides, the axial scattering force can be eliminated by constructing optical tweezers around a 4Pi microscopy, enabling the trapping of metallic nanoparticle even under the most challenging situation^17^.

However, the methods mentioned above inevitably involve the use of bulky optical elements and complicated procedures, which are difficult to be integrated into a compact platform. Surface plasmon (SP) is an electromagnetic surface wave generated at the dielectric/metal interface that caused by the interaction of metals with photons. Due to their unique characteristics such as short effective wavelength and highly spatial confinement, SPs have been widely utilized to develop miniature photonics device with dimensions much smaller than those that are currently available. Plasmonic tweezers based on the SPs excited on plasmonic structures exhibits enhanced attractive force for both dielectric and metallic nanoparticles, which provides a potential mean for manipulating nanoparticles that can be effective and flexible with a miniature device^18–20^. In this work, we propose a novel plasmonic tweezers in which a SP standing wave formed by metaholograms is applied as the trapping field. The interference of counter-propagating SPs waves gives rise to intense gradient force and zero contribution of the axial scattering force. The interaction between the nanoparticle and the SP standing wave enables not only stable three-dimensional trapping but also the precise control of the particle position by adjusting the relative phase of the illuminations. Additionally, the enhanced optical force is helpful to relieve the thermal effect, overcoming the ultimate obstacle that prevents stable trapping of resonant metallic nanoparticle^21^.

## Results

### Design of the metahologram

**Figure 1:**
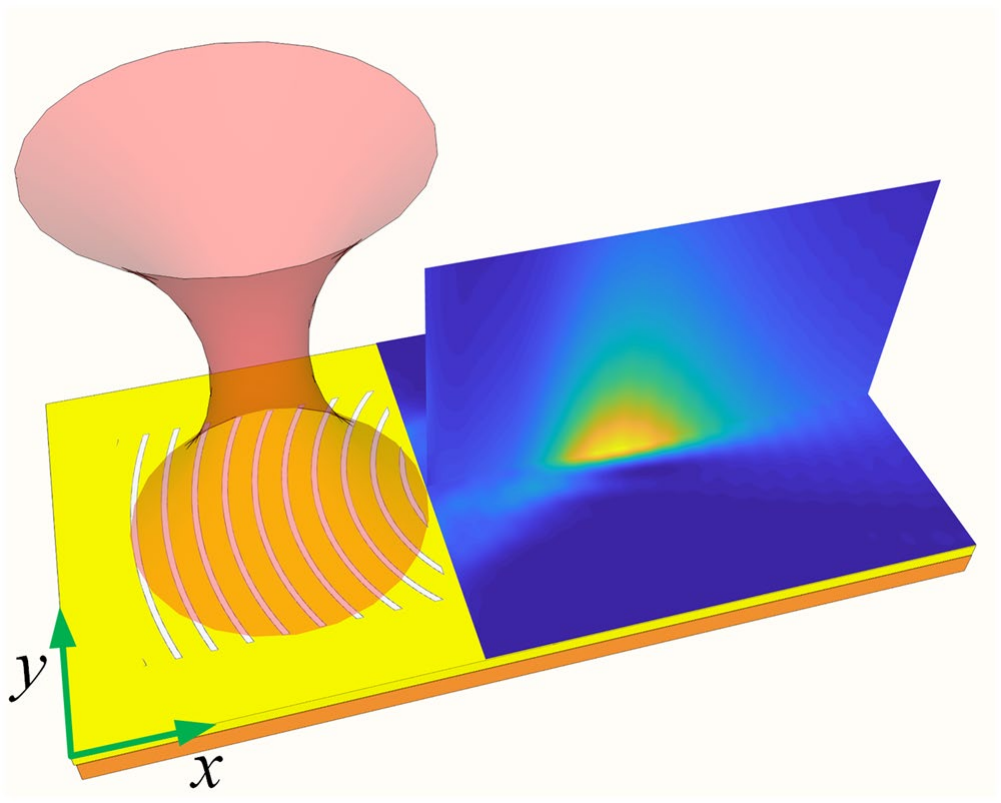
Schematic of the metahologram. The holographic pattern is designed by considering the interference between an incident beam with a converging SP wave.

**Figure 2:**
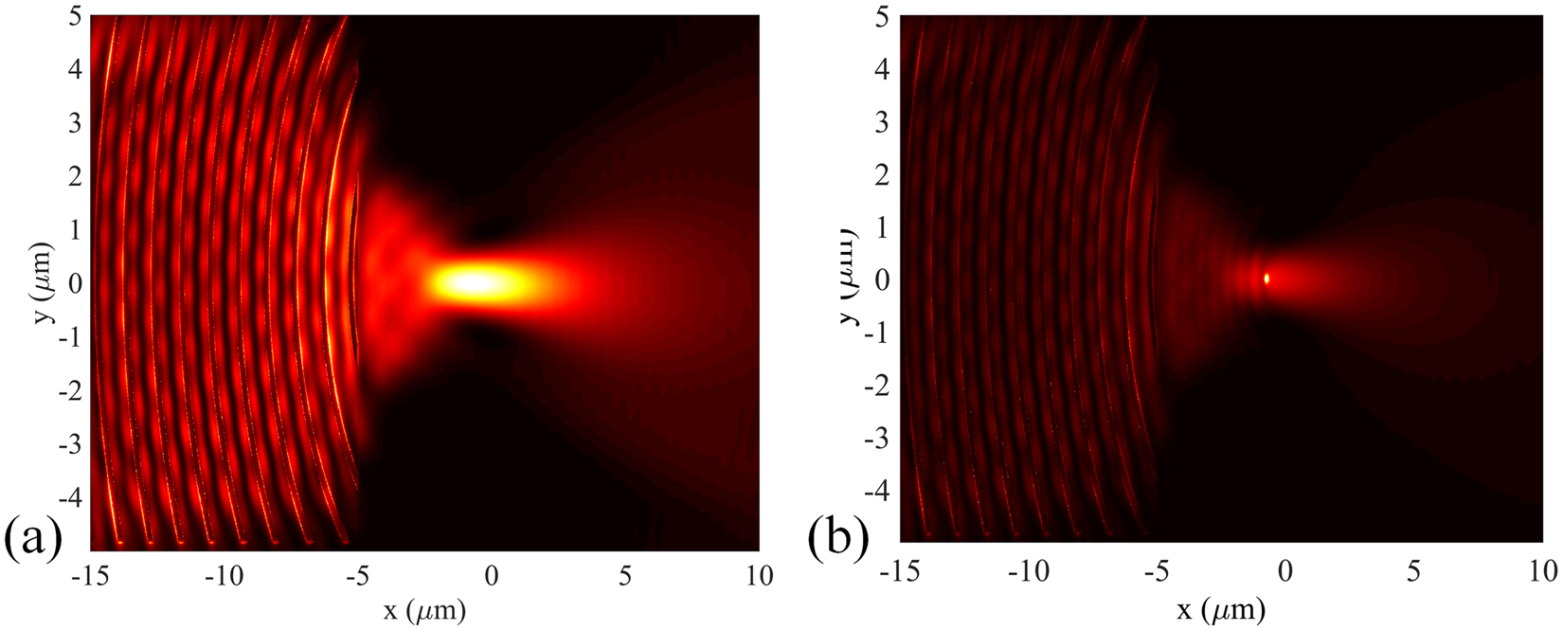
(**a**) Numerical simulation of the SP intensity distribution of the metahologram for circularly polarized illumination. (**b**) The redistribution of the SP intensity distribution when the particle locates at the peak intensity position.

Figure 1 illustrates the design of the metahologram. A holographic pattern with depth of 75 nm and area of 10 μm × 10 μm is fabricated on a gold film with thickness of 200 nm and glass substrate. This pattern is designed by interfering a converging SPs wave with incoming free-space beam^22^, which is assumed to be:

1 $${E}_{i}=A(r,z){e}^{i{l}_{g}\phi },$$

where *A*(*r*, *z*) is the transverse beam profile, *r* is the radial distance from the beam center, *φ* is the azimuthal angle, z is the axial distance from the beam waist and *l*_*g*_ is the topological charge of light also the geometrical topological charge of the hologram. To excite SPs efficiently, the period of the grooves is chosen to be 1054 nm, which is in accordance with the SPs wavelength at the gold/water interface for incident light with wavelength of 1064 nm. The non-vortex (*l*_*i*_ = 0) light with circular polarization normally shines the metahologram then be coupled into specific SP mode. It is known that the propagation direction of the SP wave depends on its topological charge (*l*_*s*_), which is determined by the conservation law of angular momentum *l*_*s*_ = *l*_*i*_ + *l*_*g*_, where *l*_g_ is the geometrical charge of the metahologram. Figure 2(a) presents the near-field intensity distribution in the focal region of the metahologram (*l*_*g*_ = 0). As expected, the illumination is coupled into focusing SP wave focal length of 5 *μ*m propagating along the axis of the metahologram.

### Optical force calculation

**Figure 3:**
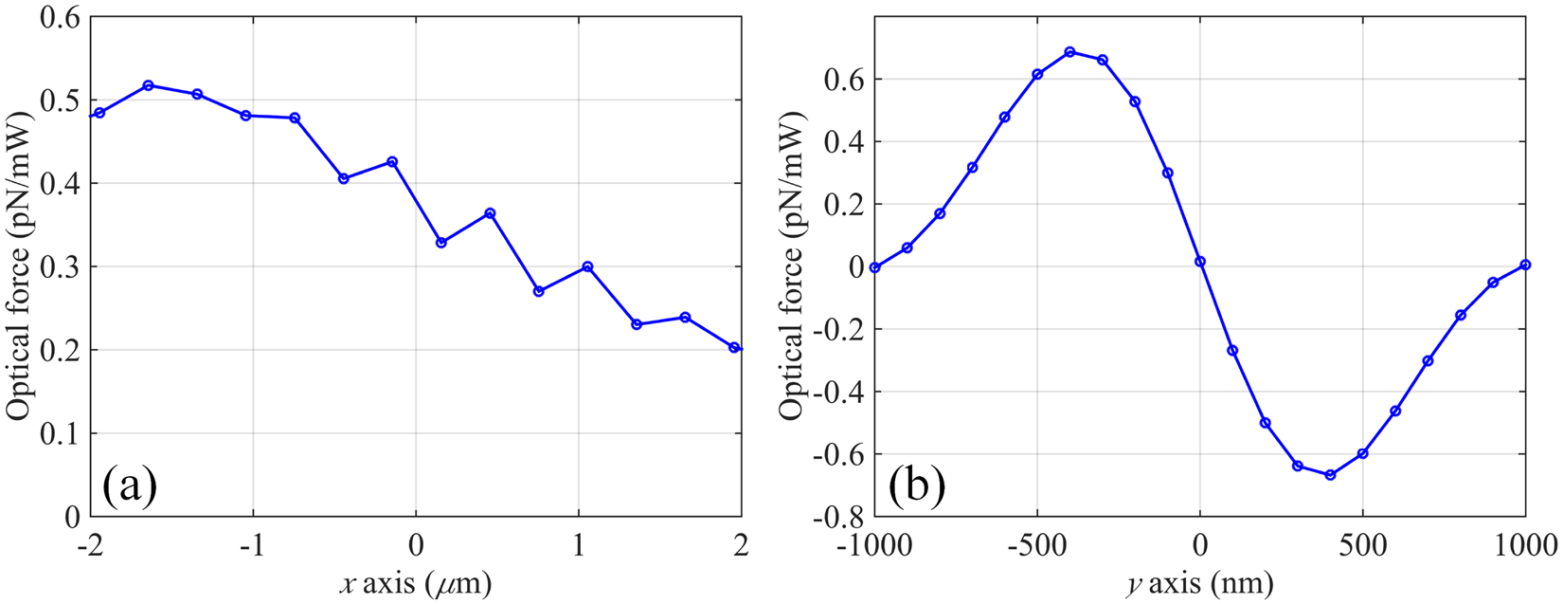
Optical force exerted on 100 nm (radius) dielectric nanoparticle along (**a**) x- and (**b**) y-axis of the metahologram.

Assuming a dielectric nanoparticle immersed in water with radius of 100 nm and refractive index of 1.59 placed within the focal region, strong scattering effect appears near the particle surface owing to the impact of the incoming optical flux (shown in Fig. 2(b)). The movement of the particle would be influenced by the exerted optical force, which can be calculated by integrating the Maxwell stress tensor (MST) over the particle surface. It is worthy of noting that the distance between the bottom of the particle and the surface of the metahologram is chosen to be 50 nm, which is a typical length for electrostatic interactions. For the MST method, the time-averaged force <F> (including both gradient force and scattering force) can be written as^23^:

2 $$\langle F\rangle =\int \{\frac{\varepsilon }{2}{\rm{Re}}[(E\cdot n){E}^{* }]-\frac{\varepsilon }{4}(E\cdot {E}^{* })n+\frac{\mu }{2}{\rm{Re}}[\mu (H\cdot n){H}^{* }]-\frac{\mu }{4}(H\cdot {H}^{* })n\}ds$$

where *ε* and *μ* are the relative permittivity and relative permeability of the medium around the particle, and *n* is the unit normal perpendicular to the integral area *ds*. The electric and magnetic field components required in the MST method are obtained directly from the FDTD simulation data. Figure 3 shows the distribution of optical force exerted on the nanoparticle in the plane parallel to the metahologram surface. Note that the forces are normalized by the injected power. Although there is an equilibrium position on the y-axis, the gradient force along the optical axis is relatively weak because of the small axial intensity gradient. Consequently, the particle would be pushed away from the metahologram by the dominating axial scattering force. In the plane vertical to the metahologram surface, the particle would be attracted to the surface due to the evanescent nature of SP.

### Configuration of the plasmonic tweezers

**Figure 4:**
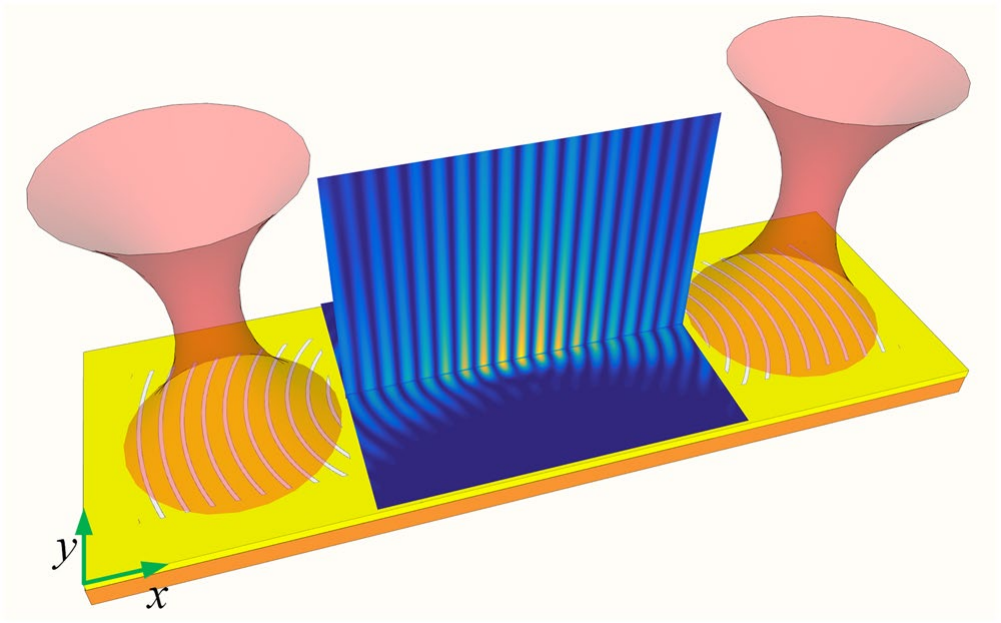
Diagram of the plasmonic tweezers consists of two identical metaholograms.

**Figure 5:**
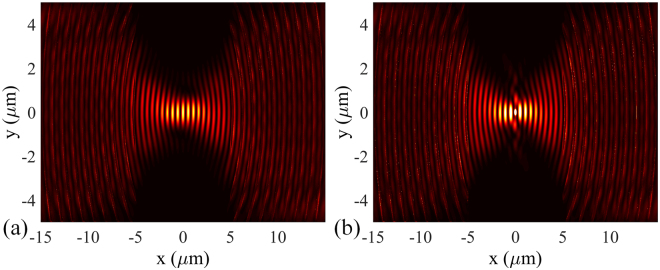
(**a**) Numerical simulation of the SP intensity distribution of the plasmonic tweezers for circularly polarized illuminations with *π* phase difference. (**b**) The redistribution of the SP intensity distribution when the particle locates at the center of the focal region.

**Figure 6:**
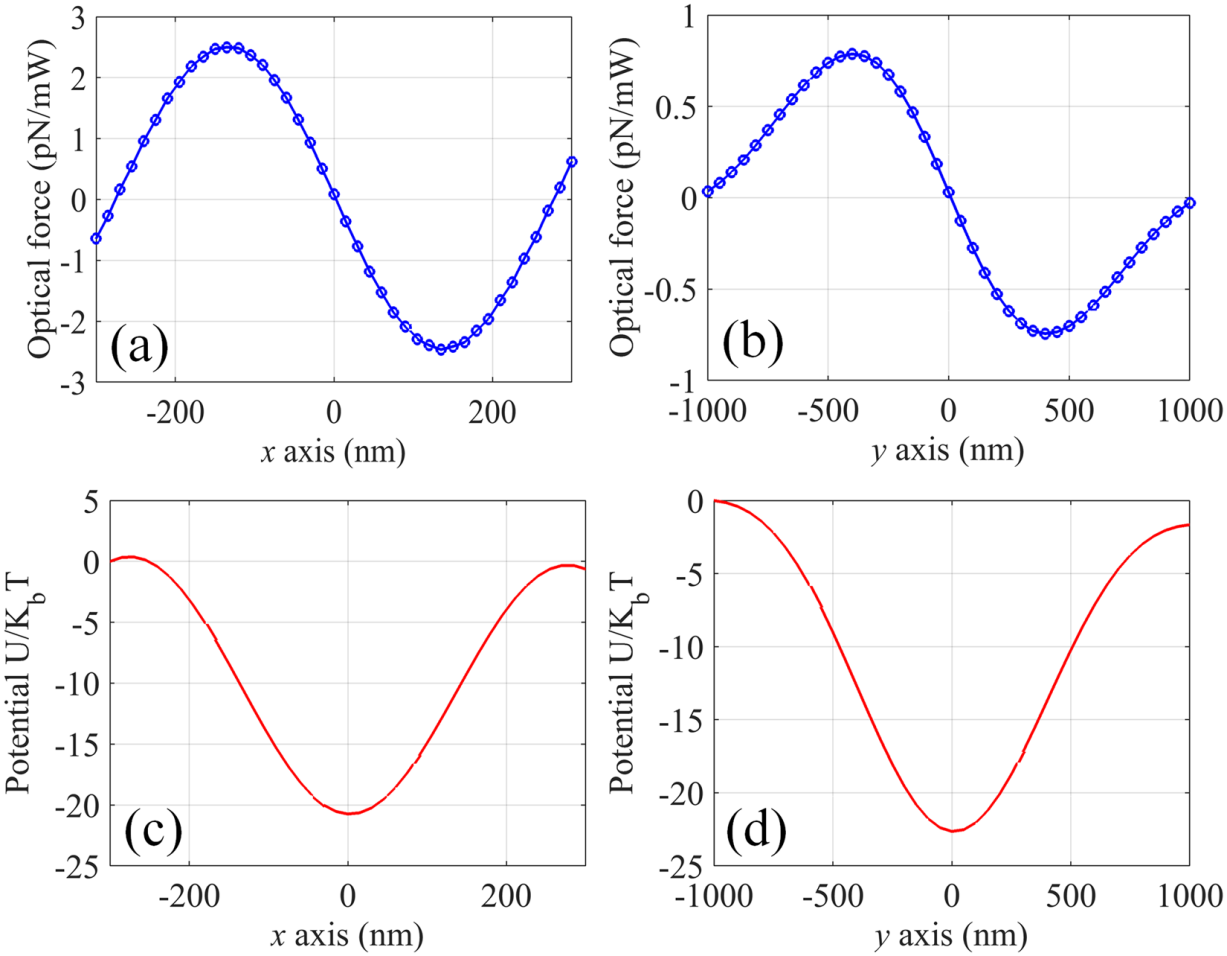
Optical force exerted on 100 nm (radius) dielectric nanoparticle along (**a**) x- and (**b**) y-axis of the plasmonic tweezers working at 1064 nm. Distribution of the corresponding potential depth along (**c**) x- and (**d**) y-axis for total input power of 200 mW.

**Figure 7:**
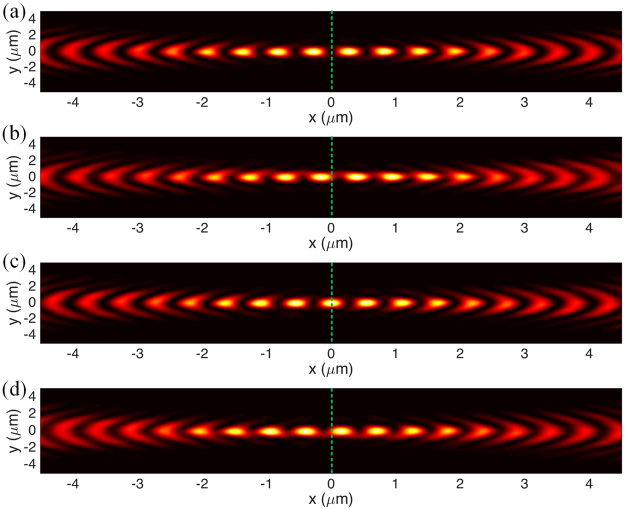
Intensity distribution of the SP standing wave when the phase difference of the illuminations is (**a**) 0, (**b**) *π*/2, (**c**) *π*, and (**d**) 3*π*/2.

In order to create an equilibrium point on the x-axis, a plasmonic tweezers consists of two identical metaholograms with separation of 10 *μ*m is proposed to balance the axial optical scattering force (shown in Fig. 4). The plasmonic tweezers is illuminated by two circularly polarized lights with phase difference of *π*. It can be seen that a SP standing wave pattern is formed by the interference of two counter-propagating SP waves (shown in Fig. 5). Assuming the dielectric nanoparticle is placed near the central focusing spot, the corresponding optical force distributions are calculated and shown in Fig. 6(a),(b). Clearly, equilibrium positions are created at both x- and y-axis. Note that the strength of the SP standing wave would become stronger by shorting the separation distance of the metaholograms, leading to further enhanced optical force. In order to evaluate the stability of the plasmonic tweezers, potential depths are estimated by integrating the optical force along the trapping direction $U=-\int F\cdot ds$. Traditionally an optical trap with potential depth larger than 1*k*_*B*_ *T* can be considered as stable, where the temperature *T* is taken to be 293 K. With the assumption that the power of each illumination is 100 mW, the maximal potential depths are calculated to be 21 × *k*_*B*_ *T* and 23 × *k*_*B*_ *T*, respectively along x- and y-axis (shown in Fig. 6(c),(d)), demonstrating the realization of the stable trap in three-dimensional space. Besides, it is known that the location of the standing wave nodes depends on the phase difference between the incident lights. Figure 7 shows the evolution of the standing wave nodes locations when the phase difference of the incident lights varying from 0 to 2*π*. The movable nodes provide a pathway to precisely control the locations of trapped nanoparticles. Note that the range of the tunability is determined by the half wavelength of the SP wave in the focal region of the metahologram.

### Trapping and manipulation of metallic nanoparticle

**Figure 8:**
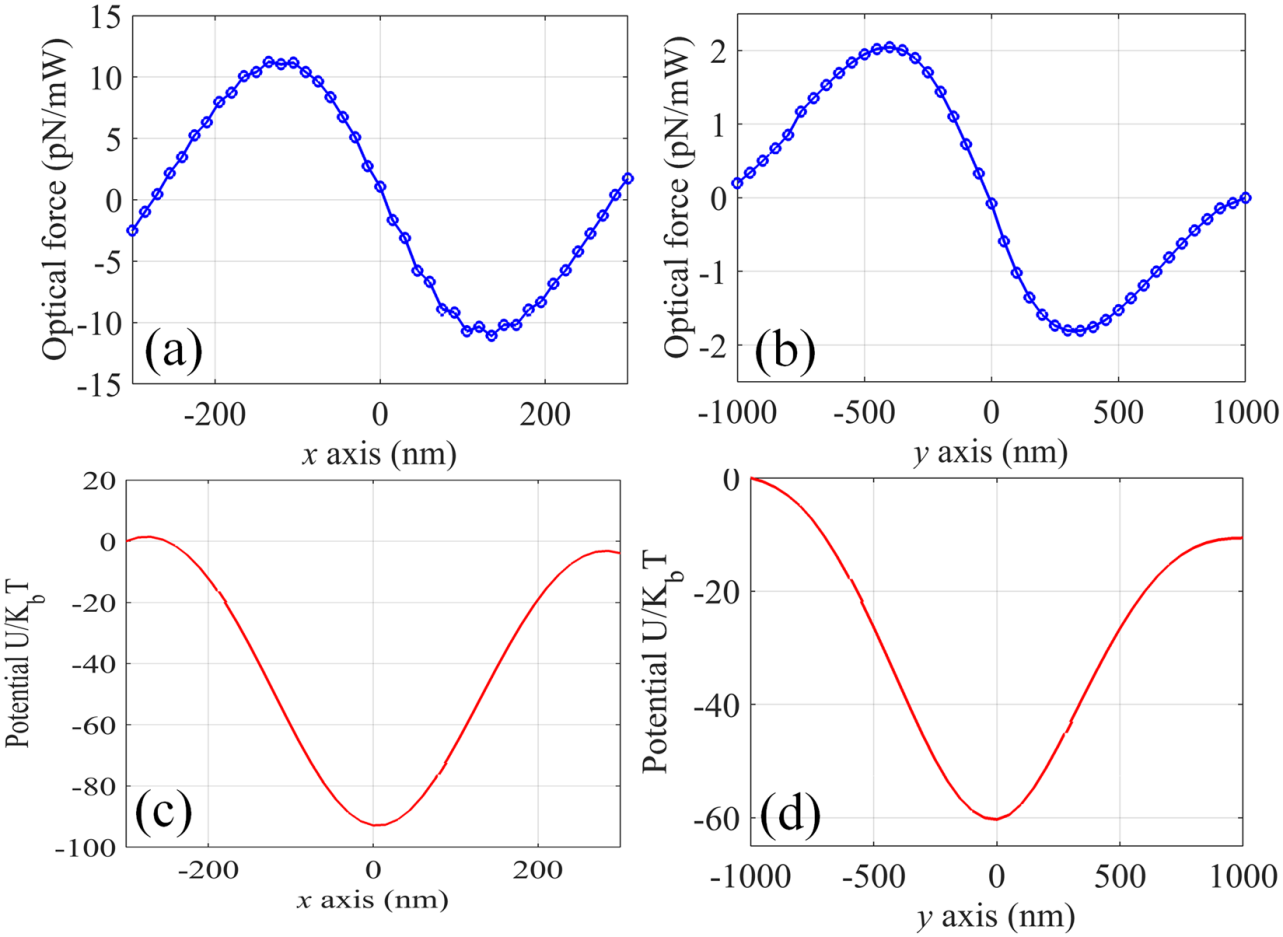
Optical force exerted on 100 nm (radius) gold nanoparticle along (**a**) x- and (**b**) y-axis of the plasmonic tweezers working at 1064 nm. Distribution of the corresponding potential depth along (**c**) x- and (**d**) y-axis for total input power of 200 mW.

**Figure 9:**
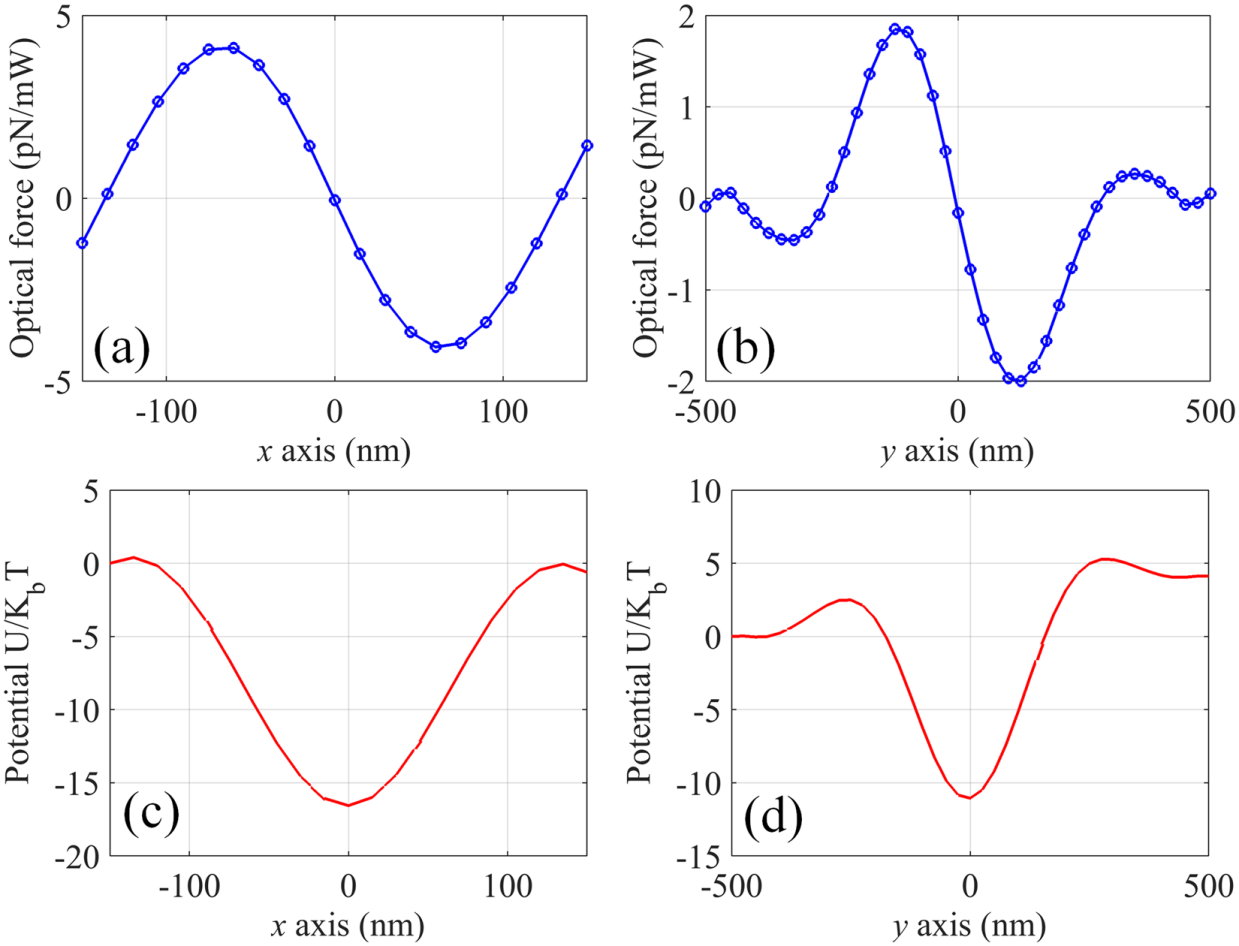
Optical force exerted on 100 nm (radius) resonant gold nanoparticle along (**a**) x- and (**b**) y-axis of the plasmonic tweezers working at 532 nm. Distribution of the corresponding potential depth along (**c**) x- and (**d**) y-axis for total input power of 200 mW.

Compared with the conventional single beam optical trap, the proposed plasmonic tweezers utilize two SP waves to create several solid focusing spot with diffraction-limit size, providing the enhanced gradient force and the possibility of manipulating multiple nanoparticle simultaneously. Moreover, the behavior of the nanoparticle along the optical axis is primarily determined by the gradient force since the axial scattering force would be canceled by the counter-propagating light wave. This advantage is especially desirable for trapping plasmonic nanoparticles, which is generally considered difficult due to the increasing scattering force. Assuming a gold nanoparticle immersed in water with radius of 100 nm placed near the central focal spot of the plasmonic tweezers, the induced optical forces and potential depth are calculated and shown in Fig. 8. Clearly a stable trapping of metallic nanoparticle can be achieved in the near-infrared region. To better illustrate the capability of the proposed plasmonic tweezers, trapping resonant metallic nanoparticle is also considered, which is the most challenging situation due to both the strong scattering force and the severe thermal effect. Considering the gold nanoparticle with resonant wavelength around 532 nm, plasmonic tweezers working at this specific wavelength is designed by following the same procedure. Note that the previous gold metahologram is not suitable because its focusing effect would be deteriorated by the resonance effect. Consequently, optical tweezers consists of two silver metaholograms (5 *μ*m × 5 *μ*m size and 5 *μ*m focal length) with separation of 5 *μ*m is proposed. As shown in Fig. 9, the resonant gold nanoparticle is still not affected by the axial scattering force, and a stable trap can also be supported in three-dimensional space from the force balance point of view. As for the thermal stability, the temperature of the particle must be kept below 647 K^24^, otherwise the optical trap would be destroyed due to the formation of vapor bubble. To study the heating effect generated by the SP interference field, an optic-thermal coupling model has been built and the temperature of the resonant nanoparticle is simulated to be only 318 K with the assumption that the total input power is 200 mW. Consequently, optical overheating effect can be avoided while maintaining large enough trapping potential, enabling stable trap of metallic nanoparticle even at the resonant wavelength.

## Conclusions

In summary, we propose a plasmonic tweezers that is capable of trapping and manipulating different kinds of nanoparticles. To trap nanoparticles in the horizontal direction, a SP standing wave is generated by illuminating two identical metaholograms. Due to the absence of the axial scattering force, the counter-propagating SP waves are feasible to trap metallic nanoparticles even under the resonant condition. More importantly, the high focusing efficiency of the metahologram enables a stable optical trapping with relatively low input power, avoiding the overheating effect that may destabilize the trap. Besides, the position of the nanoparticle can be adjusted precisely by changing the relative phase difference of the illuminations. Such technology can be easily adapted for other kinds of metallic and semiconductor nanoparticles, opening up new avenues for optical manipulation and their applications in various field.

## Methods

### Simulation method

The full-wave simulations of the characteristics of the devices shown in Figs 1 and 4 were performed using Lumerical FDTD solutions. The hologram patterns were numerically calculated with MATLAB then imported into Lumerical FDTD solutions. The optical forces were calculated by the Optical force MST toolbox available in Lumerical FDTD solutions.
